# Diagnostic Challenges in Intracranial Rosai-Dorfman Disease: Differentiating It From Meningiomas Using Imaging

**DOI:** 10.7759/cureus.82091

**Published:** 2025-04-11

**Authors:** Jad F Mourad, Mark Bryniarski

**Affiliations:** 1 Department of Neuroscience, Burrell College of Osteopathic Medicine, Las Cruces, USA

**Keywords:** adc, dwi, intracranial, meningioma, mri, neurosurgery, rosai-dorfman

## Abstract

Rosai-Dorfman disease (RDD) is a rare non-Langerhans cell histiocytosis (non-LCH) with intracranial involvement being exceptionally uncommon. We report a 45-year-old woman presenting with neurological symptoms initially suggestive of a stroke, later found to have a dural-based lesion resembling a meningioma. MRI revealed a mixed T1 isointense to hyperintense and T2 hyperintense mass with restricted diffusion on diffusion-weighted imaging (DWI) and lower apparent diffusion coefficient (ADC) values, findings atypical for meningiomas. Intracranial RDD commonly mimics meningiomas radiographically but can be distinguished by unique imaging features. Surgical resection remains the treatment of choice for isolated lesions, though recurrence is possible. This case highlights the importance of recognizing RDD in the differential diagnosis of dural-based lesions to ensure accurate diagnosis and tailored management.

## Introduction

Rosai-Dorfman disease (RDD) is a rare non-Langerhans cell histiocytosis (non-LCH) first reported in 1959 by Destombes and recognized as a clinicopathologic entity by Rosai and Dorfman in 1969. The classical or nodal form of RDD is described as a self-limited benign condition presenting with massive painless lymphadenopathy sometimes accompanied by fevers, night sweats, and weight loss [1]. RDD is frequently observed in young adults with a mean age of 20.6 years, but has been reported in the aged 1-74 years old [2]. It has increased prevalence in males and people of African descent.

In 1990, Foucar et al. [3] published the RDD registry consisting of 423 cases, with the majority presenting with extranodal disease. The most common sites of involvement in descending order of frequency were the skin, nasal cavity, paranasal sinuses, eyelid, orbit, salivary glands, and central nervous system (CNS). With a prevalence of one in 200,000 and an estimated 100 new cases per year in the United States, 43% of RDD cases present with extranodal involvement [2]. Of those, RDD of the CNS occurs less than 5% of the time, with a similar sexual distribution as the nodal form and a mean age of 39.4 years [2]. This form of RDD occurs without lymphadenopathy and usually presents with focal neurological deficits associated with the location of the mass lesion along with more general neurological symptoms, such as headaches, seizures, and gait abnormalities that evolve over weeks to months.

RDD is diagnosed through visualization and immunohistochemical staining of large histiocytes found within the affected tissue. Morphological characteristics include large round nuclei, with engulfment of inflammatory cells (emperipolesis). In nodal RDD, these histiocytes proliferate and cause expansion of the sinuses within the nodes. In contrast, fewer lesional histiocytes are visualized in extranodal RDD, with the majority of the tissue showing variable amounts of fibrosis and inflammatory infiltrate [1]. Immunohistochemical staining for RDD histiocytes shows expression of S100 and CD68 with no expression of CD1a [2].

## Case presentation

A 45-year-old woman, with a history of uncontrolled hypertension, arrived at our emergency department with a systolic blood pressure of 210 mmHg. While driving, she experienced paresthesias in her left upper extremity and face, which rapidly progressed to a generalized tonic-clonic seizure. EMS activated the stroke protocol, and she was transported to our hospital for further evaluation.

**Figure 1:**
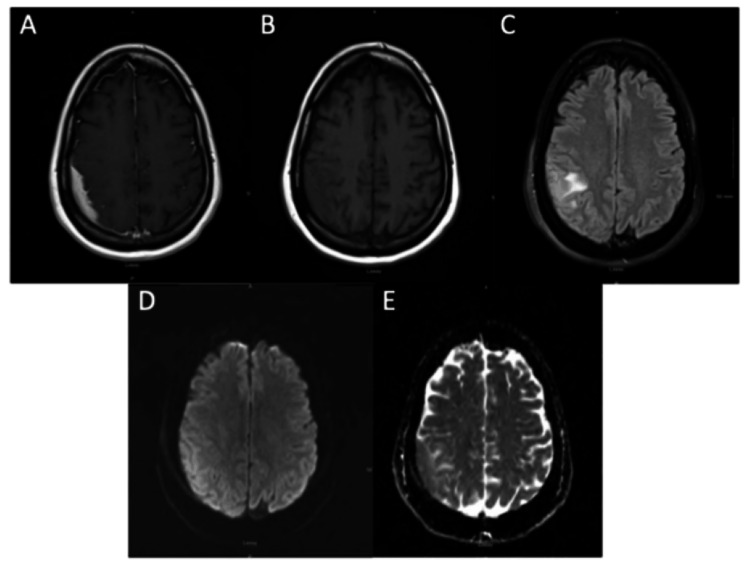
Multiple MRI sequences revealing extra-axial collection within right parietal convexity (A) Contrast-enhanced axial MRI showing homogeneously enhancing extra-axial collection within the right parietal convexity. Mixed isointense to hyperintense signals were appreciated on T1 (B) with slight hyperintense signals on T2 (C) with surrounding vasogenic edema, DWI hyperintensity (D), and ADC isointensity (E).

Workup, including a head CT without contrast, did not reveal any evidence of ischemic changes. Initial MRI of the brain without contrast showed a mixed T1 isointense to hyperintense and T2 slightly hyperintense extra-axial collection measuring 9 mm uniformly in thickness within the right parietal convexity. Mild mass effect was noted on adjacent brain parenchyma with cortical and subcortical edema with DWI hyperintensity and ADC isointensity. Subsequent MRI of the brain with and without contrast revealed a homogeneously enhancing collection with dural attachments and an irregular inferior border towards the brain parenchyma. Underlying vasogenic edema and reactive cortical thickening were present on T2-weighted sequences (Figure 1).

These findings were consistent with an intracranial meningioma in an en plaque configuration and therefore was determined to qualify for resection by craniotomy. Intraoperatively, the mass was visualized through the dura due to its discoloration. Dural opening was made sharply, the mass was identified, and careful dissection ensued. Sections were then sent to pathology for examination.

**Figure 2:**
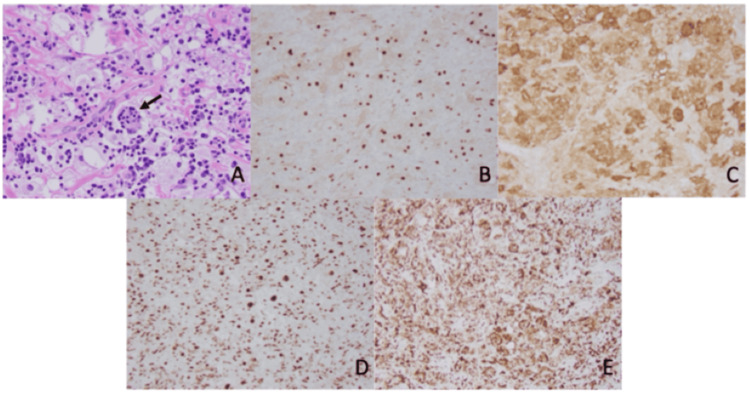
Microscopic examination (100x) of the tissue specimen revealing Rosai-Dorfman disease The specimen from dural resection showed sheets of large pale histiocytes with numerous intermixed small lymphocytes, plasma cells, and emperipolesis (A). Nuclear and cytoplasmic staining of histiocytes was positive for cyclin D1 (B), S100 (C), OCT2 (D), and CD68 (E).

Initial examination of frozen sections revealed no evidence of neoplastic tissue or elements of hemorrhage. Subsequent examination of permanent sections revealed sheets of large pale histiocytes with numerous intermixed small lymphocytes and plasma cells. Emperipolesis was present and accentuated by BCL-2 stain. Histiocytes stained positive for CD68, S100, OCT2, subset cyclin D1 (Figure 2), and negative for CD1a, langerin, BRAF, Factor XIIIa, anaplastic lymphoma kinase (ALK), and glial fibrillary acidic protein (GFAP). The background lymphocytes included a mixture of CD3+ T-cells and CD20+ B-cells. Overall, the morphologic and immunohistochemical profiles were consistent with RDD.

Postoperatively, the patient had an unremarkable recovery with no recurrence of her presenting symptoms. She was discharged in stable condition with a referral to hematology-oncology for further evaluation and management of possible systemic involvement. Given the rarity of intracranial RDD, long-term follow-up with biannual imaging was recommended to monitor for recurrence.

## Discussion

RDD of the CNS is a rare and challenging clinical entity, representing less than 5% of all RDD cases, with approximately 75% of these occurring intracranially and the rest in the spine [4]. Clinically, CNS-RDD typically presents in the absence of lymphadenopathy or constitutional symptoms, and patients often exhibit focal neurologic deficits due to mass effect. These may include seizures, headaches, cranial nerve palsies, or visual disturbances, depending on lesion location [4,5]. Zhang et al. described a 12-patient cohort of CNS-RDD in which lesions most commonly involved the cerebral convexity, parasagittal, parasellar, and petroclival regions - locations frequently implicated in previous case series as well [4,6]. The etiology of RDD remains elusive, although a number of hypotheses have been proposed. Recent studies have identified potential roles for somatic mutations in genes such as KRAS and MAP2K1, or germline variants such as SLC29A3 in familial cases, pointing toward a molecular basis in at least a subset of patients [4]. Despite this progress, no unified pathogenic mechanism has been established.

Radiologically, intracranial RDD can be difficult to differentiate from meningiomas and other dural-based tumors. Most lesions exhibit homogeneous post-contrast enhancement and may display a dural tail sign. However, certain features may raise suspicion for RDD over neoplasm. T2 hypointensity, which was present in 64% of cases in Tyagi et al.'s cohort, is believed to reflect dense collagen or inflammatory infiltrate [5]. Cheng et al. also noted this finding and proposed that it may be related to macrophage-derived free radicals or the dense cellularity of the lesion [7]. Our case demonstrated T2 hyperintensity with surrounding edema, which is less common but has been reported (Figure 1). Diffusion-weighted imaging (DWI) and apparent diffusion coefficient (ADC) mapping can be useful adjuncts in distinguishing RDD from other intracranial lesions. Cheng et al. found a mean ADC value of 0.81 × 10⁻³ mm²/s for intracranial RDD, reflecting as hypo- or isointense, less than that of meningioma (0.94). Luna et al. noted that most intracranial RDD lesions in their cohort did not demonstrate restricted diffusion on DWI, making this an uncommon finding in their experience. In contrast, our patient exhibited restricted diffusion with low ADC values, suggesting that diffusion characteristics may vary depending on histologic subtype, degree of fibrosis, or lesion location (Figure 1) [7,8].

MR spectroscopy (MRS) has also been used to characterize intracranial RDD and may offer additional diagnostic clues, demonstrating reduced N-acetylaspartate (NAA) and elevated choline-an MRS profile more typical of lesions with inflammatory or proliferative characteristics rather than overt malignancy [6,8]. However, MRS findings are not pathognomonic and should be interpreted within the broader clinical and radiologic context. Given these overlapping features with meningioma, the rate of preoperative misdiagnosis is high. Tyagi et al. found that more than half of patients in their institutional series were initially diagnosed radiologically as meningiomas [5]. Similarly, Zhang et al. reported that all nine of their convexity/skull base cases were mistaken for meningioma preoperatively [4]. The absence of calcification, hyperostosis, sunburst bony changes, and T2 hypointensity may assist radiologists and clinicians in identifying atypical lesions that warrant histologic confirmation.

Histopathologic evaluation remains the definitive diagnostic modality. The hallmark finding in RDD is emperipolesis - the presence of intact lymphocytes or plasma cells within histiocyte cytoplasm. These histiocytes are positive for S100 and CD68 and negative for CD1a, helping distinguish RDD from other histiocytoses, particularly Langerhans cell histiocytosis [4,5,7]. However, emperipolesis may be less prominent in intracranial cases due to fibrotic changes and requires careful inspection. In our case, histopathology confirmed the diagnosis with classic immunophenotypic markers and patchy emperipolesis (Figure 2).

Surgical resection remains the primary mode of treatment for intracranial RDD, especially when lesions are symptomatic or pose significant mass effect. Gross total resection (GTR) has been associated with low recurrence rates and excellent outcomes in multiple studies. Tyagi et al. reported a 24% GTR rate in their series, and Zhang et al. found that six of seven patients who underwent GTR had no recurrence during long-term follow-up [4,5]. Our patient underwent successful GTR with resolution of symptoms and no recurrence on short-term imaging. When complete resection is not feasible - such as in skull base or multicentric disease - subtotal resection, followed by adjuvant therapy, may be required. In Zhang et al.'s series, corticosteroids alone were insufficient in some cases, but combined regimens including cytarabine, lenalidomide, methotrexate, and vinblastine led to remission or disease stability in patients with recurrence or residual disease [4]. Tyagi et al. reported that 76% of patients received adjuvant therapy, often guided by lesion location and accessibility [5]. Cohen Aubart et al. further recommend considering targeted MEK inhibitor therapies for refractory or disabling cases of CNS-RDD, especially those with MAPK pathway alterations [9].

There is no established follow-up protocol for CNS-RDD, but long-term surveillance with contrast-enhanced MRI is recommended. Vaidya et al. recommend comprehensive, multimodality imaging in all patients diagnosed with RDD at a single site, as their series demonstrated that most patients had subclinical involvement of additional organ systems, including the skeleton, lymph nodes, and skin [10]. This approach helps identify multifocal disease early, guide staging and management, and monitor for progression or recurrence across nodal and extranodal compartments. Special attention should be paid to DWI and perfusion imaging, which may help distinguish recurrence from postoperative changes. While the disease is generally considered indolent, recurrence has been documented years after initial treatment, underscoring the need for vigilance even in asymptomatic patients.

## Conclusions

RDD is a rare form of non-LCH that seldom presents within the cranial cavity. MRI is the preferred imaging modality for detection, but most intracranial RDD lesions are initially misdiagnosed as meningiomas. While both pathologies share radiographic similarities, RDD often appears as a mixed isointense to hyperintense mass on T1 and homogeneously hyperintense on T2 with mild vasogenic edema. DWI can further aid in differentiation, as RDD shows can restrict diffusion with lower ADC values, unlike typical meningiomas. However, even DWI and ADC findings have been shown to vary based on other aspects of these lesions. These imaging distinctions highlight the importance of recognizing RDD in the differential diagnosis of dural-based lesions to ensure accurate identification and appropriate management.
